# Research Progress and Emerging Directions in Stimulus Electro-Responsive Polymer Materials

**DOI:** 10.3390/ma17174204

**Published:** 2024-08-25

**Authors:** Zifeng Jin, Xiaoyan Wei, Xiaojun He, Zhenglin Wang, Zhibo Zhao, Huan He, Ya’nan Yang, Nan Chen

**Affiliations:** 1 Key Laboratory of Cluster Science, Ministry of Education of China, Key Laboratory of Photoelectronic/Electrophotonic Conversion Materials, School of Chemistry and Chemical Engineering, Beijing Institute of Technology, Beijing 100081, China; 13069674955@163.com (Z.J.);; 2 Yangtze Delta Region Academy, Beijing Institute of Technology, Jiaxing 314019, China; 3 Tangshan Research Institute, Beijing Institute of Technology, Tangshan 063000, China; 4 College of Materials Science and Engineering, Beijing University of Technology, Beijing 100124, China

**Keywords:** stimuli-responsive materials, stimulus electro-responsive, polymer, moisture electric generator

## Abstract

Stimulus electro-responsive polymer materials can reversibly change their physical or chemical properties under various external stimuli such as temperature, light, force, humidity, pH, and magnetic fields. This review introduces typical conventional stimulus electro-responsive polymer materials and extensively explores novel directions in the field, including multi-stimuli electro-responsive polymer materials and humidity electro-responsive polymer materials pioneered by our research group. Despite significant advancements in stimulus electro-responsive polymer materials, ongoing research focuses on enhancing their efficiency, lifespan, and production costs. Interdisciplinary collaboration and advanced technologies promise to broaden the application scope of these materials, particularly in medical and environmental protection fields, ultimately benefiting society.

## 1. Introduction

Smart responsive polymer materials possess the remarkable ability to respond to various external stimuli and subsequently undergo reversible changes in certain physical or chemical properties. These materials can react to a diverse range of stimuli, including temperature fluctuations, light exposure, mechanical forces, humidity levels, pH changes, electric fields, and magnetic fields [1,2,3,4,5,6]. Electroactive smart materials can rapidly respond to external stimuli, making them highly useful in sensors and detectors. Additionally, their ease of miniaturization and integration facilitates their application in wearable devices and enables high-output performance for various applications. Owing to their high energy conversion efficiency, electroactive smart materials also play a significant role in energy harvesting. Utilizing these materials to fabricate sensors presents a promising application area, enabling health monitoring when worn on the body, medical diagnostics in the healthcare sector, and environmental condition monitoring [7,8,9,10,11,12]. One of the most notable characteristics of stimulus electro-responsive materials is their ability to maintain good responsiveness over multiple cycles of exposure to these stimuli. This cyclic stability ensures that their performance remains consistent and reliable even after repeated activation and deactivation. The dynamic nature of these materials allows them to adapt and respond precisely to environmental changes, making them highly valuable for a wide array of applications. Their ability to reversibly alter their properties in response to different stimuli positions them as key components in the development of advanced sensing technologies, adaptive systems, and energy harvesting devices [13,14,15,16,17,18]. The ongoing research and innovation in the field of stimulus electro-responsive materials continue to expand their potential applications, driving progress in various scientific and industrial domains. In the early 20th century, scientists discovered that certain materials exhibited different properties under changes in temperature or light, marking the first research into stimulus electro-responsive materials. By the mid-20th century, scientists discovered that some polymer materials could also change their properties in response to external stimuli. Since then, polymer materials have been widely studied as a key type of stimulus electro-responsive material [13,14,15,19]. Later, researchers developed polymers that respond to multiple stimuli, such as photo-thermal, thermo-magnetic [20], and thermo-mechanical [21], which can generate electrical responses to various types of stimuli [22,23,24,25,26]. In recent years, humidity-stimulated electro-responsive materials have been rapidly developing and have become a new trend in the evolution of smart responsive materials [27]. This technology utilizes humidity electro-responsive materials to directly generate electrical signals through interaction with ubiquitous environmental moisture, further advancing the development of smart materials in multifunctional response directions.

In the existing literature, the property of generating non-specific responses (such as deformation, temperature change, luminescence, or electrical signals) to non-specific external stimuli is termed “stimuli-responsive” [28,29,30,31]. Materials exhibiting this property are referred to as “stimuli-responsive materials” or “smart materials” [32,33]. However, the primary focus of this review is on materials that can generate electrical signal responses, or at least include electrical signal responses, when subjected to various external stimuli. This means that regardless of the type of stimulus the material is subjected to, the resulting voltage and current electrical signal responses are the focus of our concern. The relationship between these two categories is that “materials capable of generating responses to external stimuli” include “materials that have electrical signal responses to external stimuli”. Since the properties of the objects described in this review are a subset of “stimuli-responsive”, using “stimuli-responsive” to describe the research subjects of this review would not be accurate. However, the existing literature lacks a specific term for the property of generating electrical signal responses to non-specific external stimuli. Therefore, we designate this property as “stimulus electro-responsive”, meaning the ability to produce electrical signal responses such as current and voltage under external stimulus conditions (e.g., thermal, optical, humidity stimuli). Materials with this property can be applied in sensor fields where environmental changes need to be perceived and corresponding electrical responses are required, outputting the relevant electrical signals. It is important to distinguish this property from “electro-responsive”. “Electro-responsive” refers to the ability to generate some form of response under the influence of an electrical signal or electric field, i.e., inputting electrical energy and outputting other forms of energy [34,35,36,37,38]. In contrast, “stimulus electro-responsive” involves inputting other forms of energy and outputting electrical energy. Given that the primary focus of this review is on polymers, we will refer to polymers that produce electrical signal responses under external stimuli as “stimulus electro-responsive polymers”.

## 2. Conventional Stimulus Electro-Responsive Polymer Materials

When exposed to various external stimuli, polymer materials can generate electrical signal responses. Thermal stimulus electro-responsive polymer materials, for instance, respond to temperature changes by producing electrical signals. Similarly, photo stimulus electro-responsive polymer materials generate electrical signals under specific light conditions. Additionally, there are polymer materials that exhibit electrical signal responses when subjected to mechanical force, as well as those that respond electrically when exposed to magnetic fields. In this section, we discuss representative traditional stimulus electro-response polymer materials, outlining their unique properties and mechanisms for generating electrical signals under different external stimuli. 

### 2.1. Thermal Stimulus Electro-Responsive Polymer Materials

Thermal stimulus materials can typically produce electrical signals when their temperature changes. Inorganic thermal stimulus materials are usually costly and inefficient, while organic conductive polymers often have low thermal conductivity, are easy to synthesize and process, and are low-cost, making them promising for research in thermal stimulus devices. Organic conductive polymer–inorganic thermoelectric (TE) composites also have the advantages of low cost and high performance [39]. In TE materials, holes and electrons spontaneously diffuse from the high-temperature side to the low-temperature side under a temperature gradient, generating voltage and detectable current in an external circuit. Among thermal stimulus materials, PEDOT:PSS (Poly(3,4-ethylenedioxythiophene):poly(styrene sulfonate)) has been one of the most successful materials in recent years. Equation 1 shows that the figure of merit (ZT) is commonly used to characterize the performance of a TE material, where σ is the electrical conductivity, κ is the thermal conductivity, S is the Seebeck coefficient, and T is the absolute temperature. The product of σ and S2 represents the power factor (PF = σS2), which is often used to measure the performance of TE materials [40].

(1) $$\mathrm{ZT}=\frac{S^{2}\sigma T}{\kappa }$$

**Figure 1:**
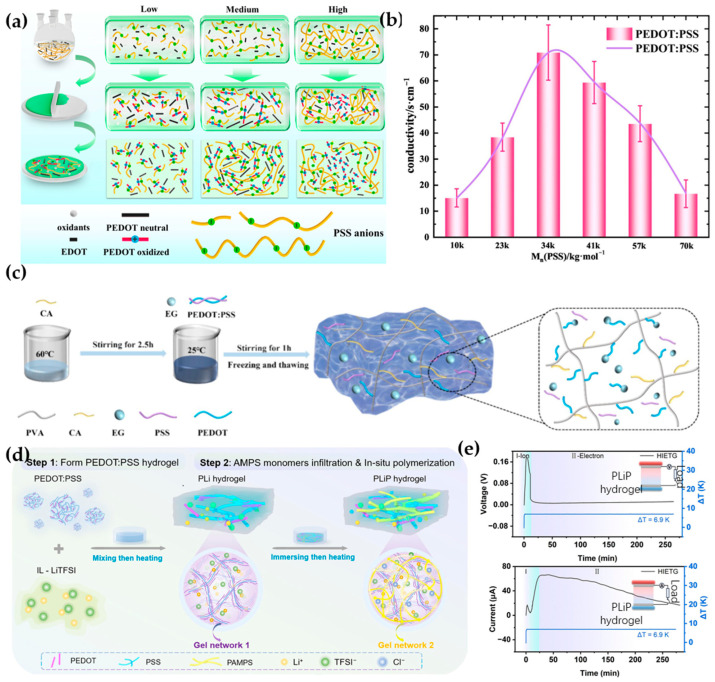
Synthesis and performance of PEDOT TE materials. (**a**) Schematic illustration of the mechanism by which the molecular weight of PSS affects PEDOT [42]. (**b**) Impact of PSS molecular weight on electrical conductivity [42]. (**c**) Synthesis of hydrogel and the distribution of citric acid and ethylene glycol within the hydrogel [43]. (**d**) Schematic of hydrogel preparation [44]. (**e**) Voltage and current generation time during practical testing of the hydrogel [44].

PEDOT:PSS is a polymer material that responds to multiple stimuli. Since Jiang et al. first investigated the TE properties of PEDOT:PSS [41], researchers have begun to study the TE properties of composites containing PEDOT:PSS. Fan et al. found that the molecular weight of PSS affects the TE properties of PEDOT:PSS (Figure 1a), with the electrical conductivity of PEDOT:PSS first increasing and then decreasing as the molecular weight of PSS increases (Figure 1b) [42]. Zhu et al. used PEDOT:PSS as a sensing material and polyvinyl alcohol (PVA) hydrogel as the matrix, simultaneously adding citric acid and ethylene glycol (Figure 1c) to improve the mechanical properties and antifreeze performance of the hydrogel. This sensor can sense temperature through voltage changes and strain through resistance changes, with the advantages of high sensitivity, low detection limit, good stability, and fast response and recovery times [43]. To address the low TE efficiency caused by the intermittent utilization of heat in ionic thermoelectric (i-TE) technology, Zhang et al. combined i-TE materials with electronic thermoelectric (e-TE) materials. The team used lithium bis(trifluoromethanesulfonyl)imide (LiTFSI) to dope PEDOT:PSS, forming gel network 1, and then doped poly(2-acrylamido-2-methyl-1-propanesulfonic acid) (PAMPS) with chloride anion (Cl^−^), forming gel network 2, introducing an interpenetrating network structure into the material to improve conductivity (Figure 1d) and achieving a TE performance of 7.86 mV/K. The hydrogel can generate electricity continuously for over four hours (Figure 1e), reaching an energy density of 93.7 J/m^2^ [44]. In addition to PEDOT:PSS, other organic polymers also exhibit good TE properties. Liu et al. used electrochemical treatment in an [EMIM]BF4 ionic liquid (IL) to alter the doping state of polythiophene (PTh) films, increasing the power factor and electrical conductivity by 3.15 times and 1.3 times, respectively, compared to the original films [45]. 

**Figure 2:**
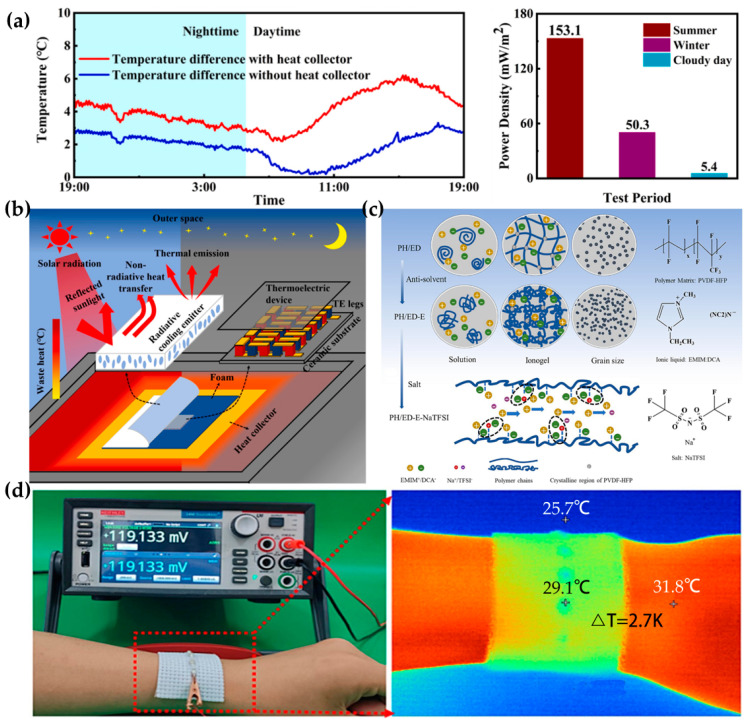
Performance of RCE with wristband TEGs. (**a**) Variation in the temperature difference across the TEG with the addition of a heat transfer plate [46]. (**b**) Schematic of a micrometer polymer film utilizing building waste heat [46]. (**c**) Addition of ethanol and NaTFSI to the PVDF-HFP/EMIM, PH/ED ion gel [48]. (**d**) High voltage generation with a temperature difference of only 3 K [48].

Zhang et al. added a heat transfer plate to thermoelectric generators (TEGs), expanding the temperature difference between the two ends of the TEG by 2.3 times and achieving an output power of 153.1 mW/m^2^ (Figure 2a). The team used microporous poly(vinylidene fluoride-co-hexafluoropropylene) (P(VdF-HFP)) micro-thick polymer films as radiative cooling emitters (RCEs), successfully utilizing building waste heat (Figure 2b) [46]. Malik et al. added silica nanoparticles to polyaniline, creating a poly(2-acrylamido-2-methyl-1-propanesulfonic acid):phytic acid (PANI:PAAMPSA:PA) ternary polymer, enhancing the self-healing ability and stretchability of TEGs while increasing the TE efficiency to an unprecedented level (ZT = 3.74) [47]. Some research teams have developed TEGs for human applications, requiring TEGs to be flexible and capable of generating power at low temperature differences. In a poly(vinylidene fluoride-co-hexafluoropropylene)/1-ethyl-3-methylimidazolium dicyanamide (PVDF-HFP/EMIM:DCA PH/ED) ionic gel, the addition of ethanol and sodium bis(trifluoromethylsulfonyl)imide (NaTFSI) (Figure 2c) improved ion mobility, allowing the TEGs to output high voltage even in high-humidity environments. Wearing a wristband made of this material in an environment with a temperature difference of less than 3 K can still generate 0.12 V of high voltage (Figure 2d) [48]. Because i-TE materials have Seebeck coefficients two to three orders of magnitude higher than e-TE materials, designing a solid network to immobilize ILs in ionic gels can improve the output performance of TEGs by enhancing ion conductivity. Adding PVDF-HFP to EMIM:DCA increased the ion gel’s electrical conductivity by 2.5 times, raising the ZT value to 1.8 [49]. Duan et al. report a novel concept that enables p-n conversion for the iodide/triiodide (I^−^/I_3_^−^) redox couple induced by poly(N-isopropylacrylamide) (PNIPAM) thermosensitive nanogels, with the Seebeck coefficient changing from 0.71 mV/K to −1.91 mV/K. The nanogels enable selective capture of I_3_^−^ at the hot side, followed by the release of I_3_^−^ at the cold side, yielding a concentration gradient of the free I_3_^−^ and resulting in p-n inversion. The wearable device developed by this team harnessed body heat to generate an open-circuit voltage of 1 V and an output power of 9 µW [50]. Wang et al. introduced PNIPAM as a thermo-responsive component and selected polyaniline (PANI) as the conductive component. The resulting topologically co-crosslinked hydrogels can change their resistance by altering the temperature or the bending angle, thereby adjusting the current flow. The response time can reach 0.4 s, and the sensing stability is maintained for at least 350 cycles. This system can be further applied to monitor human motion, including hand movements, joint bending, and even swallowing, and pulse rate [51]. Zhang et al. utilized the photothermal conversion properties of PANI and the temperature responsiveness of conductive ILs to prepare conductive thermosensitive inks. These PANI/ILs inks can be easily screen-printed onto various flexible substrates to create sensors. Under infrared light irradiation, the chip temperature increases, and the conductivity of the IL changes, achieving thermosensitivity in PANI/IL-based chips. The device’s temperature varies with different infrared light power levels due to differences in heat absorption efficiency, resulting in varying rates of current change and enabling sensitive responses to heat and light exposure [52].

### 2.2. Light-Stimulus Electro-Responsive Polymer Materials 

**Figure 3:**
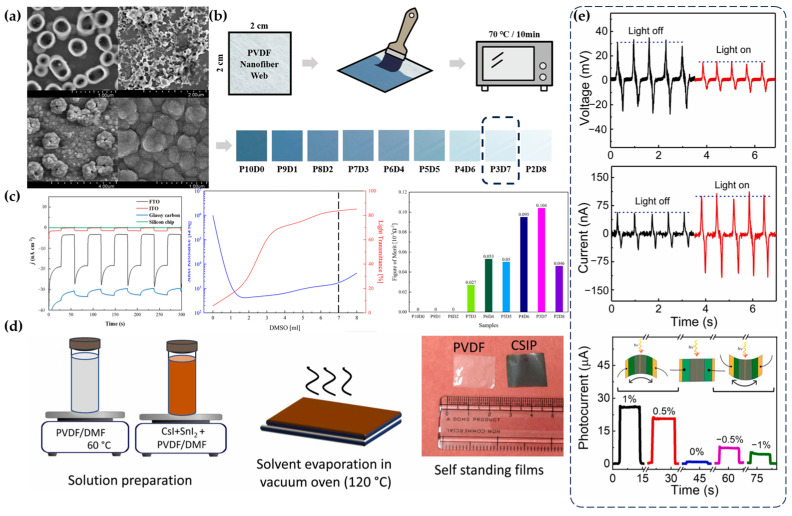
Preparation and performance of light-stimulus electro-responsive polymer materials. (**a**) Different morphologies and performances of films deposited on four different substrates [54]. (**b**) Schematic of TCE preparation using brush-coating technology [55]. (**c**) Impact of DMSO content on resistance and transmittance, and the effect of PEDOT and DMSO content on performance [55]. (**d**) Schematic of MHPs incorporated into PVDF films [56]. (**e**) Material exhibiting photoelectric effect and piezo-phototronic effect [56].

Under light conditions, some polymer materials can generate electrical signals using the photoelectric effect or photovoltaic effect. When the energy of the photons illuminating the material is sufficiently large, electrons on the material’s surface are excited to form photoelectrons, or photons generate electrons and holes inside the material. Under the influence of an electric field, electrons and holes are separated and move in different directions, with electrons flowing through electrodes to the external circuit to generate current. PTh-based polymers are among the most widely used materials for light-stimulus power generation. The fundamental principle of power generation in PTh-based polymers can be explained as follows: under illumination, electrons inside the material are excited, diffusing to the interface where charge separation occurs, and finally, charges are collected at the electrodes. The efficiency of these processes determines the electrical output performance [53]. Gao et al. used electropolymerization to deposit poly(1,3,5-tris(thiophen-2-yl)benzene) (PTTB) on different conductive substrates, forming PTTB films with different morphologies. They found that the morphology of the films significantly affected the photoelectric performance (Figure 3a) [54]. Cha et al. used brush-coating technology to create dye-sensitized textile-based transparent conductive electrodes (TCEs) using PEDOT:PSS/PVDF nanofibers (Figure 3b). They explored the optimal ratio of PEDOT:PSS to dimethyl sulfoxide (DMSO) to achieve maximum light transmittance and minimum sheet resistance (Figure 3c). The test results showed that the electrical output could reach 73.2 mV of voltage and 0.44 mA/cm^2^ of current density [55]. Currently, most light-stimulus materials are inorganic compounds, so much of the research on light-stimulus materials involves doping inorganic materials with photoelectric effects into conductive polymers that can function as nanogenerators, using the photoelectric effect as an auxiliary function for piezoelectric or triboelectric nanogenerators. For instance, Mallick et al. added metal halide perovskites (MHPs) to PVDF films (Figure 3d) to produce the piezo-phototronic effect (Figure 3e) [56]. Tavakoli et al. creatively used monolayer hexagonal boron nitride deposited by chemical vapor deposition as an electron-blocking layer in organic photovoltaics. Due to the large barrier hexagonal boron nitride poses to electrons and the small barrier it poses to holes, it can block electrons while creating possible paths for holes to move to the electrode [57]. Ferrocene can also be used as an additive in polymers to enhance their photoelectric properties. Mehta and colleagues developed a series of all-polymer thin-film systems by incorporating dibutyl ferrocene polymer electrolytes or poly(N-methylpyrrole) ferrocene donor layers and octyl ferrocene polymer electrolyte acceptor layers. These systems exhibited long-lasting photovoltage phenomena, maintaining an output of nearly 700 mV even after 30 min of exposure to light with a wavelength of 450 nm [58].

### 2.3. Mechanical-Stimulus Electro-Responsive Polymer Materials

Materials may produce electrical signals when subjected to mechanical stimuli, with piezoelectric nanogenerators (PENGs) and triboelectric nanogenerators (TENGs) being common examples. 

**Figure 4:**
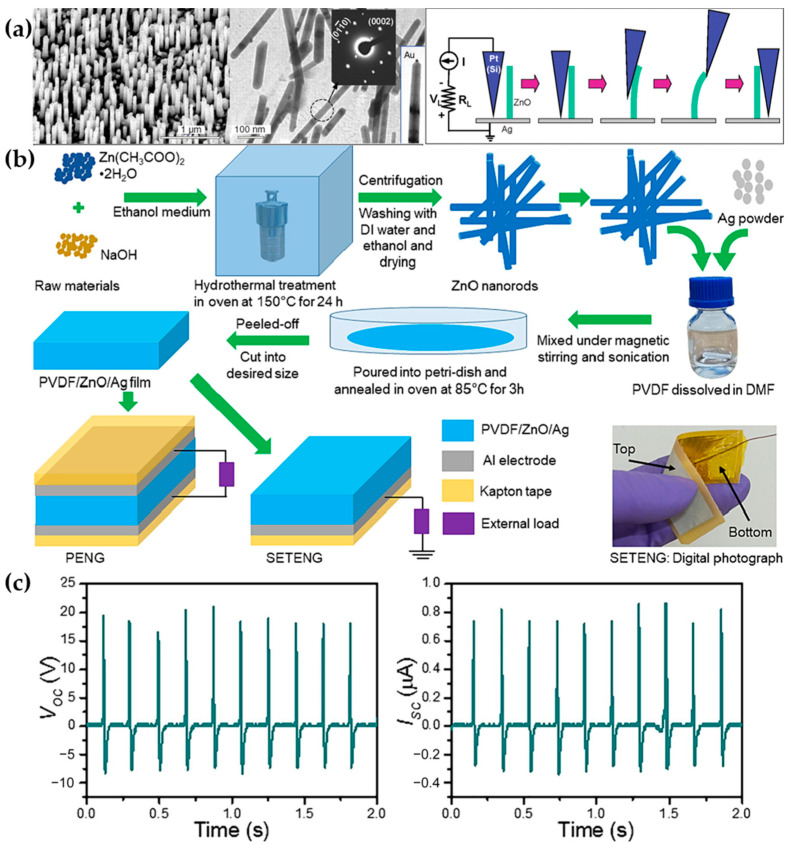
Preparation and performance of piezoelectric polymer materials. (**a**) First observation of piezoelectric phenomenon in zinc nanowires [59]. (**b**) Preparation process of PVDF/ZnO/Ag and the structure of the PENG device [61]. (**c**) Open-circuit voltage up to 28 V and short-circuit current of 1.2 μA [61].

The principle of PENGs is based on the piezoelectric effect, where materials generate charge separation and form a potential difference under pressure. Piezoelectric materials often use nanomaterials with a large specific surface area, such as nanowires (Figure 4a), to produce significant charge separation under small external forces [59]. Among organic polymers, PVDF has the best piezoelectric properties. Under the action of external forces, its molecules undergo dipole changes, generating a potential difference [60]. Although the piezoelectric properties of PVDF are good, they are still slightly inferior to those of certain inorganic materials. Therefore, the most common approach is to dope inorganic materials into the PVDF substrate to enhance the output performance of PENGs. Sasmal et al. developed a composite material, PVDF/ZnO/Ag (Figure 4b), which has good biocompatibility and can be used directly on the human body. PENGs made from this material can output an open-circuit voltage of 28 V and a short-circuit current of 1.2 μA (Figure 4c), showing potential for medical detection applications [61].

**Figure 5:**
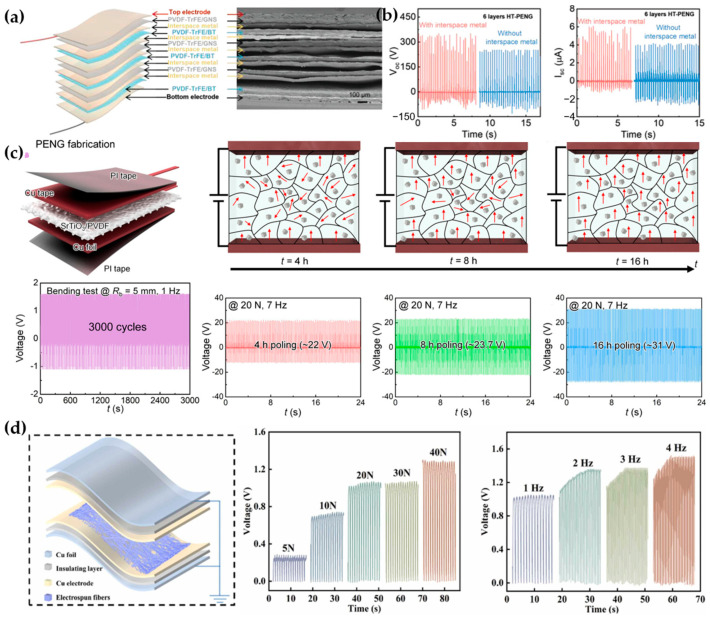
Innovative piezoelectric structures and materials and their performance. (**a**) Schematic and SEM images of the heterogeneous structure piezoelectric device [62]. (**b**) Output performance of the heterogeneous structure piezoelectric device [62]. (**c**) Schematic of the PVDF piezoelectric material structure with titanate additives and its piezoelectric performance [63]. (**d**) Schematic of the testing device structure and voltage output under different conditions [64].

Mahanty et al. combined piezoelectric composite nanofibers with heterostructure and interstitial metal sheets to enhance stress concentration (Figure 5a), dispersing the received external force well. The composite nanofibers in the material not only increased the piezoelectric coefficient but also reduced the impedance of the material, directly enhancing the output performance of PENGs (Figure 5b) [62]. Adding titanate or other inorganic substances into PVDF or PVDF copolymers can also significantly improve the piezoelectric properties of the materials (Figure 5c,d) [63,64,65,66,67].

**Figure 6:**
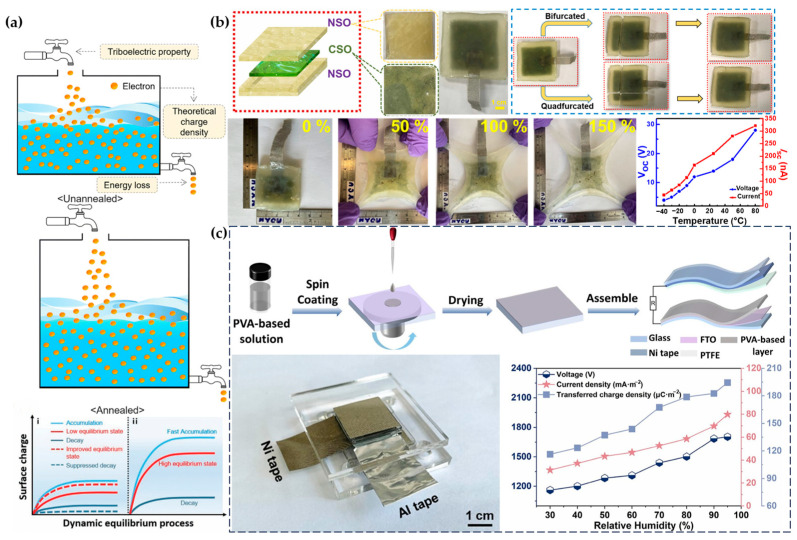
Preparation processes and performance of different piezoelectric materials. (**a**) Schematic of the accelerated charge injection process reducing leakage charge [69,70]. (**b**) Supermolecular gel with stretchability and self-healing ability and its electrical output performance [71]. (**c**) TENG functioning properly under high-humidity conditions [72].

The principle of TENGs is based on the triboelectric effect and electrostatic induction. When two different materials come into contact, electrons transfer from the less electronegative material to the more electronegative material. When the materials separate, their surfaces retain opposite charges, forming an electrostatic field. Connecting the two materials through an external circuit can generate current [68]. Increasing the charge density on the material surface is an effective method to improve the performance of TENGs. Some studies have proposed ultra-fast charge injection technology and accelerated charge accumulation processes to increase surface charge density (Figure 6a) [69,70]. Khan et al. prepared supramolecular gels with strong tensile properties and rapid self-healing capabilities, which can operate normally at temperatures ranging from −40 °C to 80 °C (Figure 6b) [71]. Wang et al. added LiCl and MXene to PVA films with moisture absorption properties, successfully creating TENGs with high output performance even in high-humidity conditions (Figure 6c) [72]. Having full self-healing ability can extend the lifespan of TENGs and expand their application range [71,73]. Mariappan et al. used polycarbyne (PVDF) as the friction layer for TENGs, increasing the output to 120 V [74]. The most widespread application of TENGs is as wearable devices carried by people.

**Figure 7:**
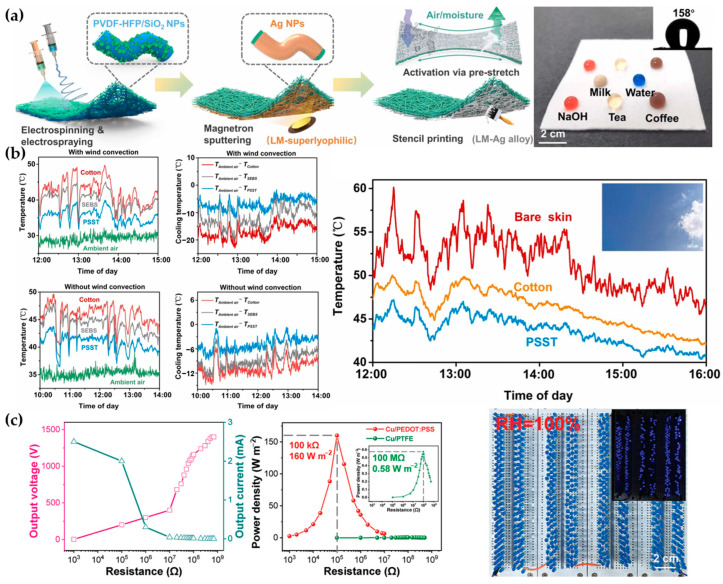
Innovative applications of piezoelectric polymer materials. (**a**) Hydrophobicity of TENG after electrospinning [75]. (**b**) TENG integrated with passive radiative cooling technology maintaining a comfortable temperature in any weather [75]. (**c**) High voltage output from PEDOT film and copper or aluminum foil as triboelectric pairs, with good performance under high humidity [77].

Compared to skin-adhered sensor devices, Fan et al. manufactured a comfortable nonwoven fabric by electrospinning poly(styrene-ethylene-butylene-styrene) (SEBS) and electrospraying PVDF-HFP/silica (Figure 7a), integrating passive radiative cooling technology into the TENG and bringing TENG devices closer to daily life applications (Figure 7b) [75]. Pan et al. added tourmaline (TM) to PVDF nanofibers, increasing the power density of TENGs by 1.5 times compared to pure PVDF TENGs, achieving an open-circuit voltage of 267 V and enhancing the output performance by 2.1 times. This TENG-based micro-capacitor has broad applications in powering portable electronic products [76]. Sun et al. used PEDOT:PSS films and copper or aluminum foil as the triboelectric pairs, achieving an impressive output of 1400 V and 1333 mA/m^2^, even operating normally under high-humidity conditions or in the presence of water droplets (Figure 7c) [77].

### 2.4. Magneto-Stimulus Electro-Responsive Polymer Materials

Certain polymer materials, known for their excellent stimulus-responsive properties, such as piezoelectric materials, urgently require enhancement through doping with other substances. This doping process is essential to augment their responsiveness to various external stimuli and to broaden their range of potential applications. By incorporating additional substances, the inherent properties of these polymer materials can be significantly improved, enabling them to exhibit heightened sensitivity and performance in response to multiple types of stimuli, including mechanical stress, electrical fields, temperature changes, and more. This enhancement not only optimizes their functional capabilities but also expands their usability in a variety of advanced technological applications. The process of doping these materials aims to integrate and amplify their piezoelectric properties, thereby making them more effective and versatile. This, in turn, facilitates the development of more efficient and robust devices for energy harvesting, sensing, and other responsive applications. Consequently, the strategic enhancement of polymer materials through doping is a crucial step in advancing their performance and extending their application horizons in the ever-evolving field of stimulus-responsive technologies. For example, adding materials with magnetoelectric coupling properties to conductive polymers enables the composite material to generate electrical signals under a magnetic field. Because the β-phase dipole moment of the copolymer poly(vinylidene fluoride-trifluoroethylene) (P(VDF-TrFE)) is the highest among conductive polymers, it has been the mainstream material in piezoelectric and triboelectric devices. Gupta et al. added magnetite (Fe_3_O_4_) nanoparticles synthesized on a micron-sized magnesium hydroxide [Mg(OH)_2_] template to P(VDF-TrFE), reducing leakage in the composite material and inducing magnetoelectric effects. The magnetoelectric coupling coefficient (αME) of this composite film was 30 mV/cm [78]. With electronic devices almost everywhere, the magnetic fields generated by electronic devices surround us. In this context, using magnetoelectric devices for daily energy harvesting has become a research focus. Nam et al. coated P(VDF-TrFE) on a magnetostrictive film made of cobalt ferrite (CoFe_2_O_4_ CFO) nanofibers and a polyimide matrix to prepare flexible magnetoelectric composites. Under an external alternating magnetic field of 1 Hz, this wearable magnetoelectric energy harvesting device can generate 0.52 V and 25 nA of electrical output, offering new possibilities for the future development of magnetoelectric devices [79].

## 3. New Directions in Stimulus Electro-Responsive Polymer Materials

**Figure 8:**
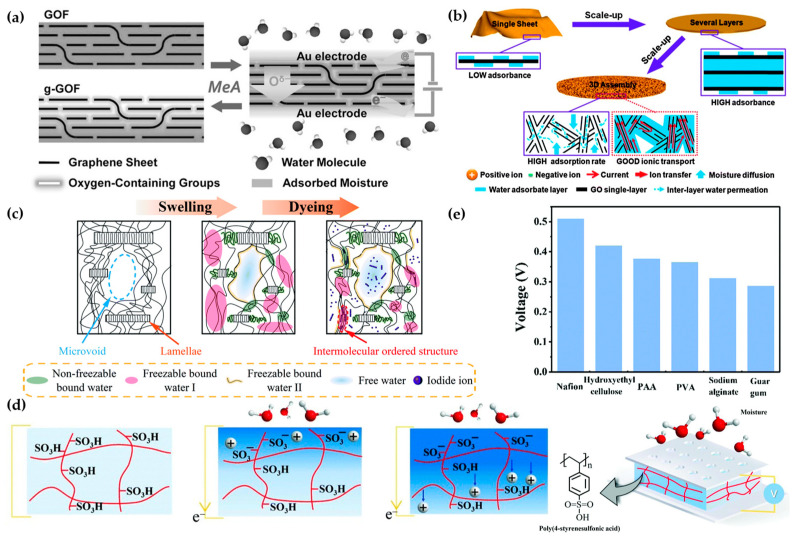
Structure and mechanism of MEGs. (**a**) Mechanism of GO MEG [27]. (**b**) Water adsorption process and ion transport process [80]. (**c**) Water adsorption and desorption process of water-absorbing materials [81]. (**d**) Power generation mechanism of sulfonic acid-containing materials under humidity [82]. (**e**) Electrical response to humidity of different water-absorbing materials [82].

Compared to the stimuli mentioned in the previous section, humidity stimuli are relatively novel and have broad research prospects. Zhao et al. used carbon materials as the first generation of humidity-stimulus electro-responsive polymer materials. They utilized graphene oxide (GO) films with a gradient of oxygen-containing functional groups that could adsorb water molecules under humidity stimuli. During adsorption, the H^+^ ions dissociated from the oxygen-containing functional groups migrate spontaneously under the concentration gradient, generating potential and current. When water desorbs from the film, it promotes the recombination of dissociated oxygen-containing functional groups and hydrogen ions, causing the GO film to return to its initial state (Figure 8a) [27]. Although many research teams have since developed different humidity-stimulus electro-responsive polymer materials, such as polymers, hydrogels, and ionogels, the basic principle of generating electrical signals under humidity stimuli remains similar to Zhao’s work. Among these, humidity stimulus electro-responsive polymer materials have emerged as a significant research hotspot in the field of stimulus-responsive materials. In addition to responding to humidity stimuli, the development of multi-stimulus responsiveness represents a new and exciting direction for polymer-based stimulus-responsive materials. Typically, a single material can be engineered to produce electrical signals in response to two or more of the previously mentioned stimuli, such as temperature, light, mechanical force, or magnetic fields. This multi-stimulus responsiveness considerably enhances the efficiency of energy collection and broadens the range of potential applications for these materials. By leveraging the ability to respond to multiple stimuli, these advanced polymer materials can be utilized in more diverse and complex environments, thereby significantly improving their utility and effectiveness in various technological applications. This dual or multifunctional capability not only optimizes the performance of energy harvesting systems but also makes these materials more versatile for use in sensors and other responsive devices. The ongoing research and development in this area highlights the potential for polymer humidity-stimulus materials to play a crucial role in the future of energy harvesting and environmental monitoring technologies.

### 3.1. Humidity-Stimulus Electro-Responsive Polymer Materials

Water is an exceptionally abundant resource on Earth, making it a vital component in various energy utilization strategies. Mainstream methods of harnessing energy from water predominantly focus on the energy contained within liquid water. However, it is important to recognize that the energy content of atmospheric water vapor is also substantial and has significant potential. Humidity-stimulus electro-responsive materials harness the energy from water vapor to generate power, effectively tapping into underutilized water energy resources. These materials have gained significant attention in recent years due to their benefits, such as low cost, high energy conversion efficiency, and wide-ranging applicability. Consequently, they have become a major focus of research. The primary application of humidity-stimulus materials is in the development of sensors capable of generating electrical signals in response to humidity levels. These sensors are instrumental in monitoring human activities or detecting environmental changes associated with air humidity. In addition to their sensing capabilities, these materials also find some applications in energy harvesting, further enhancing their versatility and utility. The integration of humidity-stimulus materials into various technological applications underscores their potential to contribute significantly to the fields of environmental monitoring and renewable energy solutions. The two most important processes for moisture electric generator (MEG) devices are adsorbing moisture from the air and asymmetric ion transport within the material (Figure 8b). Based on these two processes, polymers become highly competitive materials for humidity-stimulus applications. In the MEG power generation mechanism, a critical step is the adsorption of atmospheric moisture by the device, meaning the water absorption properties of the material significantly affect the actual performance of MEG devices [80]. Certain functional groups in polymers, such as the sulfonic acid groups in poly(4-styrenesulfonic acid) (PSSA), can strongly adsorb moisture from the air [83]. PVA also contains numerous hydrophilic functional groups, allowing it to spontaneously adsorb moisture when exposed to the air (Figure 8c) [81]. Xu et al. used PSSA to fabricate humidity-electric devices, successfully outputting an open-circuit voltage of 0.8 V and a short-circuit current density of 0.1 mA/cm^2^. The hydrophilicity of the PSSA film was used to adsorb moisture, dissociating hydrogen ions from sulfonic acid groups under the influence of water. These hydrogen ions migrate inward under the concentration gradient, generating electrical output (Figure 8d). The team tested other materials, such as PVA, polyacrylic acid (PAA), hydroxyethyl cellulose, Nafion, and natural polysaccharides, to verify this mechanism (Figure 8e) [82].

**Figure 9:**
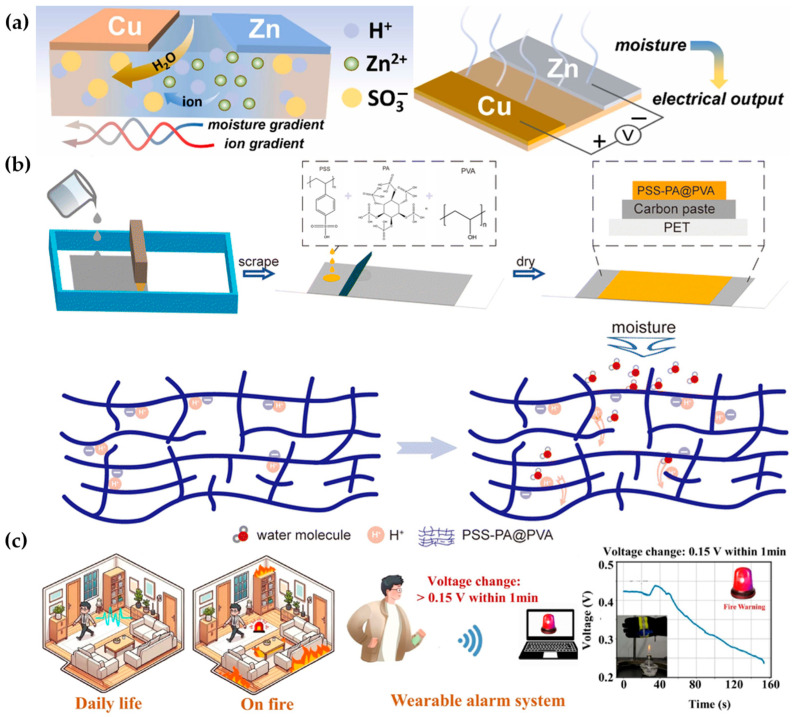
Mechanisms and applications of other MEGs. (**a**) Mechanism of ion transport in a lateral double-gradient system [84]. (**b**) Schematic of the preparation method and ion transport mechanism of MEG with added water-absorbing PA [87]. (**c**) Innovative applications of flame-retardant MEGs [88].

Feng et al. used hygroscopic polymer PSS covered with asymmetric metal electrodes to form lateral humidity gradients and ion gradients within the polymer layer. This dual-gradient mechanism significantly improved the output performance of the humidity-electric device (Figure 9a), achieving a voltage output of 1 V and a power density of 874 μW/cm^2^. Due to its fast response (0.68 s) and high dynamic range (85,700%), the device can detect various moisture levels in breath to realize real-time monitoring of human conditions [84]. This extended the direction of ion migration from perpendicular to the film surface to along the direction of the film extension. The strong hygroscopicity and high ionization ability of PA, especially when incorporated into other polymer materials, can significantly improve the ionic conductivity of materials [85,86], These properties make PA an excellent additive for MEGs. Han et al. innovatively used PSSA, PVA, and PA to create a humidity-electric device that outputs an open-circuit voltage of 0.88 V and a power density of 1.36 mW/cm^2^ (Figure 9b) [87]. Xing et al. designed a wearable, high-output-power, breathable, and flame-retardant humidity-electric device using PVA/PA and PVDF nanofiber films, achieving an output voltage of 1.0 V and a power density of 3 μW/cm^2^, greatly enriching the application scenarios of humidity-electric devices (Figure 9c) [88]. Doping inorganic materials into organic polymers can also enhance the output performance of humidity-electric devices. Duan et al. fabricated a polyacrylamide ionic hydrogel on silicon nanowire arrays, resulting in a humidity-electric device with high output voltage (1.2 V) and high output stability (maintaining 60% output performance after 800 h) [89]. Zhang et al. used sodium alginate (SA)/PEDOT:PSS to form a core–shell structure and multi-walled carbon nanotubes to enhance charge migration along fibers and axes, achieving a maximum output voltage and current of 1.2 V and 21 mA, respectively, with a power density of 5.56 W/m^2^ [90]. Feng et al. developed a composite material PU@PSS that not only generates electricity from humidity but also uses environmental heat to improve ion migration efficiency, achieving high-performance output (1.875 W/m^2^) under heating conditions [91]. Glycerol–water binary solvent-based ionic hydrogels can also enhance the performance of humidity-electric devices. Yang et al. combined PVA, PA, and the solvent system, with a single device continuously generating about 0.8 V for over 1000 h. The integrated humidity-electric device can also drive a 210 V device [92]. Huang et al. used acid-doped poly(4-styrenesulfonic acid) (H-PSS) to fabricate MEG devices using 3D printing technology. This method is not limited by the substrate, is easier to scale up, and can easily integrate devices producing over 180 V and more than 1 mA of current [93]. The mechanical properties, safety, and biocompatibility of polymer materials give them a significant lead over inorganic materials in specific MEG applications. Zhang et al. developed a humidity-electric device using waterborne polyurethane (WPU) that is water-resistant and stretchable. The WPU-based device can still generate about 0.3 V after 50 washes and 1000 stretches [94].

**Figure 10:**
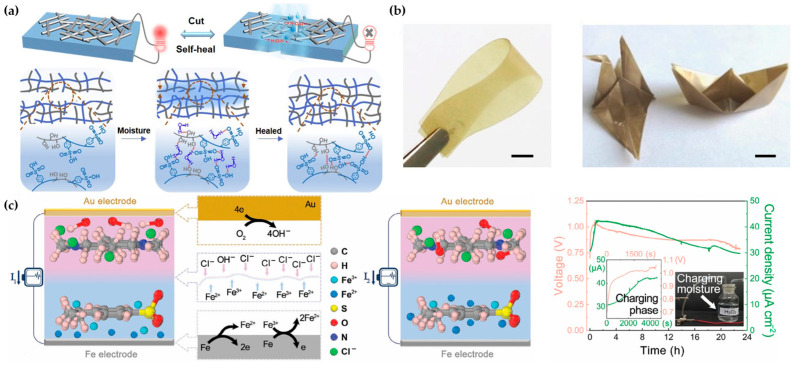
Recent innovative developments in MEGs. (**a**) The mechanism of self-healing when MEG is in a high-humidity environment [95]. (**b**) MEG possesses excellent deformability [95]. (**c**) MEG incorporating redox reactions [96].

Wang et al. developed a PSS-PVA film with self-healing capabilities (Figure 10a). This humidity-electric device can still produce high output (0.6 V, 2.0 μA) after repeated bending and can self-heal when damaged (Figure 10b), with a healing efficiency of up to 100% [95]. Recent studies have shown that introducing redox reactions into MEG devices can improve electrical output, making MEG devices evolve towards batteries. Shi et al. used PSSA/Fe^3+^ and PDDA to form dual-humidity electric functional layers, producing a humidity-electric battery with an output voltage of up to 1.08 V. The innovative introduction of Fe^3+^ caused the MEG to generate electricity through both ion migration triggered by concentration differences and redox reactions between active metal electrodes Fe and Fe^3+^, significantly improving the MEG’s output voltage (Figure 10c). This research not only enables long-term stable operation (2080 h) but also maintains nearly 100% voltage output after a hundred charge–discharge cycles [96]. Liu et al. developed a novel humidity-driven power generation device using an ionic polymer Nafion and PNIPAM hydrogel. This device can sustainably achieve an ultra-high voltage of 1.86 V with a single module, allowing it to charge commercial capacitors and electrochromic smart windows. The introduction of PNIPAM also provides a reliable additive for enhancing the output performance of the humidity-driven power generation device [97].

### 3.2. Multi-Stimulus Electro-Responsive Polymer Materials

**Figure 11:**
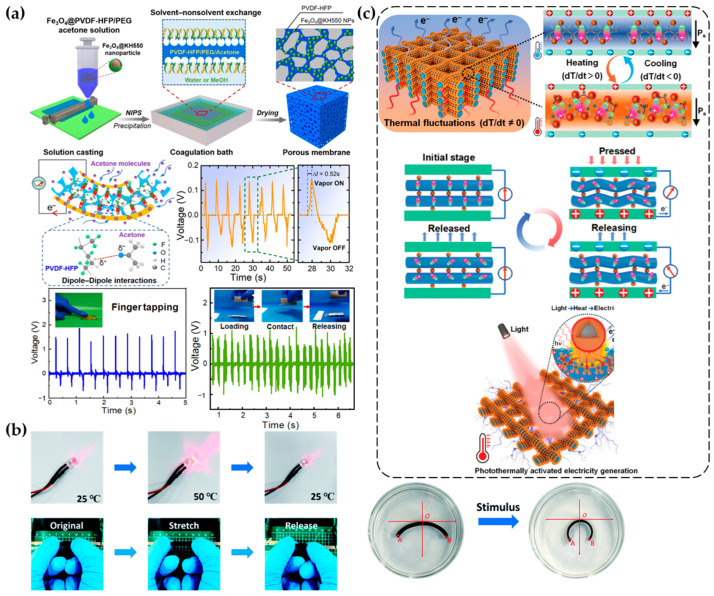
Performance of multi-stimulus electro-responsive polymer materials. (**a**) PVDF-HFP nanocomposite films doped with magnetic Fe_3_O_4_ responding to both pressure and magnetic fields [24]. (**b**) Effect of temperature on resistance, with self-bending under external stimuli [25]. (**c**) Electrical response of PNHs to light, heat, and pressure [26].

As the development of various stimulus-responsive devices progresses, researchers have discovered that certain materials possess the capability to respond to multiple stimuli rather than being limited to a single stimulus. These materials can produce electrical responses when exposed to different types of stimuli. Owing to this unique characteristic, multi-stimulus-responsive materials typically exhibit a wider range of potential applications. When utilized as energy harvesting devices, they demonstrate significantly higher output performance. Additionally, as sensors, these materials have the capability to provide comprehensive monitoring of environmental changes, making them highly versatile. The ability of these materials to simultaneously respond to multiple external stimuli positions them as highly promising candidates in the fields of energy harvesting and environmental sensing. The enhanced performance and versatility of multi-stimulus electro-responsive polymer materials indicate their substantial potential in advancing the capabilities of both energy harvesting systems and sensor technologies. For example, Li et al. developed a three-dimensional porous structure TENG of PVDF-HFP nanocomposite film (Figure 11a), which has multi-stimulus sensing capabilities. The addition of magnetic Fe_3_O_4_ nanoparticles to the TENG allows it to respond to magnetic fields. Additionally, the device can produce continuous electrical responses to organic solvent vapors. The sponge-like porous structure within the film significantly enhances the piezoelectric coefficient of the TENG [24]. Yu et al. used a double-layer hydrogel composed of poly(2-(dimethylamino)ethyl methacrylate) and poly(N-isopropylacrylamide). The hydrogel bends and deforms in response to different stimuli (temperature, pH, sodium chloride, ethanol) [25].

Adding carbon black to the hydrogel network imparts conductivity. One major advantage of this hydrogel is its flexibility, allowing it to change its bending angle with varying external conditions. The hydrogel’s resistance changes with temperature, making it suitable as a thermistor for temperature sensors, which can change the brightness of an LED lamp with temperature variations (Figure 11b) [25]. Cui et al. were inspired by muscle fibers and combined polymer nanofibers and nanoparticles through multiscale regulated interfacial electrostatic and chemical interactions to form piezoelectric polymer nanohybrid structures (PNHs). These PNHs significantly enhance piezoelectricity and exhibit TE effects under temperature changes, with TE coefficients higher than most traditional polymer materials. This material can also generate electricity under illumination (Figure 11c), with photoelectric conversion efficiency about ten times higher than traditional materials [26].

## 4. Conclusions and Outlook

This review systematically reviews the current research progress on stimulus electro-responsive polymer materials. Despite the significant achievements that have been made in terms of performance, devices fabricated from these materials still fall short of replacing conventional everyday items with similar functionalities. This shortfall is due to the fact that traditional sensor devices have reached a high level of perfection in various aspects, including manufacturing processes, cost-efficiency, safety, accuracy, sensitivity, and durability. For sensors or energy harvesting devices created from new self-powered stimulus electro-responsive materials to gradually capture market share and eventually become commonplace or even indispensable in daily life, several critical aspects must be addressed. Firstly, there is a need to develop more efficient stimulus electro-responsive materials by gaining a deeper understanding of the principles governing different stimulus responses and continuously optimizing the output performance of nanogenerators to enhance energy conversion rates. Secondly, the output performance of these devices can be improved through appropriate integration, enabling them to power devices with higher energy demands. Thirdly, extending the lifespan of nanogenerators is crucial, which can be achieved by altering material compositions to minimize the need for maintenance and replacement in practical applications. Fourthly, continuous refinement of synthesis methods and assembly processes is essential to reduce production costs and improve the utilization of raw materials. Furthermore, fostering interdisciplinary collaboration and embracing advanced technologies, such as biotechnology, are paramount. The inherent functionality of smart responsive materials endows them with significant roles in fields such as medicine and environmental protection. Active engagement in interdisciplinary collaboration will not only broaden the application range of stimulus electro-responsive materials but also increase the likelihood of these innovations benefiting humanity on a larger scale.

## Data Availability

No new data were created or analyzed in this study.
